# Ferrocene‐ and Biferrocene‐Containing Macrocycles towards Single‐Molecule Electronics

**DOI:** 10.1002/anie.201702006

**Published:** 2017-05-12

**Authors:** Lucy E. Wilson, Christopher Hassenrück, Rainer F. Winter, Andrew J. P. White, Tim Albrecht, Nicholas J. Long

**Affiliations:** ^1^ Imperial College of ScienceDept. of ChemistrySouth KensingtonLondonSW7 2AZUK; ^2^ Universität KonstanzFachbereich ChemieUniversitätsstraße 1078457KonstanzGermany

**Keywords:** alkynes, electrochemistry, ferrocene, macrocycle, spectroelectrochemistry

## Abstract

Cyclic multiredox centered systems are currently of great interest, with new compounds being reported and developments made in understanding their behavior. Efficient, elegant, and high‐yielding (for macrocyclic species) synthetic routes to two novel alkynyl‐conjugated multiple ferrocene‐ and biferrocene‐containing cyclic compounds are presented. The electronic interactions between the individual ferrocene units have been investigated through electrochemistry, spectroelectrochemistry, density functional theory (DFT), and crystallography to understand the effect of cyclization on the electronic properties and structure.

Cyclic structures have enthralling beauty that has attracted chemists for generations.^1^, ^2^ Such structures display a wide range of unusual electronic properties, not seen in linear analogues.^3^, ^4^ The inherent difficulty of synthesizing such molecules is often due to the competition between cyclization and polymerization.^5^ These difficulties produce complex synthetic routes, low yields, or restrictions in the structures that can be formed. Methods to overcome this ring/chain equilibrium include pre‐organization and dynamic supramolecular assembly.^6^, ^7^, ^8^

Communication between redox‐active centers is highly studied, with the identification of useful bridging ligands, binding groups, and suitable redox‐active components, furthermore establishing a solid understanding in redox center communication and electronic coupling in mixed valence states.^9^, ^10^, ^11^ Multiple redox‐active groups within molecular wires show interesting results in conductance modulation.^12^, ^13^, ^14^ Currently, little research has been pointed towards redox‐active groups in parallel or in rings within electronic systems.^2^, ^15^, ^16^, ^17^ Ferrocene is often incorporated into molecular wires due to its stability, high conductance levels, and ability to act as a redox switch.^18^, ^19^, ^20^, ^21^ The biferrocene motif is of additional interest, owing to the extra redox state and intervalence charge transfer. Biferrocene has been incorporated into molecular electronic wires but not yet into cyclic systems.^22^ Several ferrocene containing cyclic structures have been synthesized.^2^, ^23^, ^24^ Organization of ferrocene into such structures can be problematic due to its flexibility, creating diversity of structures. This generates synthetic challenges, which our group have overcome with specifically designed synthetic routes and utilizing diethynyl bridges to incorporate high levels of rigidity and conjugation into our design.^17^, ^25^, ^26^, ^27^ Herein, we discuss synthetic routes for the incorporation of three and four ferrocene units into a covalently bonded cyclic conjugated system, enabling investigation into the interaction of the neighboring redox‐centers and the effects of variation in symmetry.

Cyclic compounds can be synthesized by several different methods. We devised a route to the triferrocene cyclic system through a stepwise assembly to a covalently bonded system (Scheme 1). 1,1′‐Diiodoferrocene was synthesized by a recently developed, improved procedure and coupled with 1,3‐diethynylbenzene using a Sonogashira cross‐coupling with Pd(P*t*Bu_3_)_2_ as the catalyst.^2^ Within our group, this catalyst has been found to show superior results for coupling alkynes with ferrocene due to the large cone angle of the P*t*Bu_3_ ligand.^28^ Favorable yields of 25 % of the desired product **1** over polymeric side products were achieved through the use of a 10:1 excess of 1,1′‐diiodoferrocene to alkyne. Compound **1** was purified by column chromatography, also allowing the retrieval of the excess of 1,1′‐diiodoferrocene (see the Supporting Information, Figure S2 for NMR spectra).

**Scheme 1:**
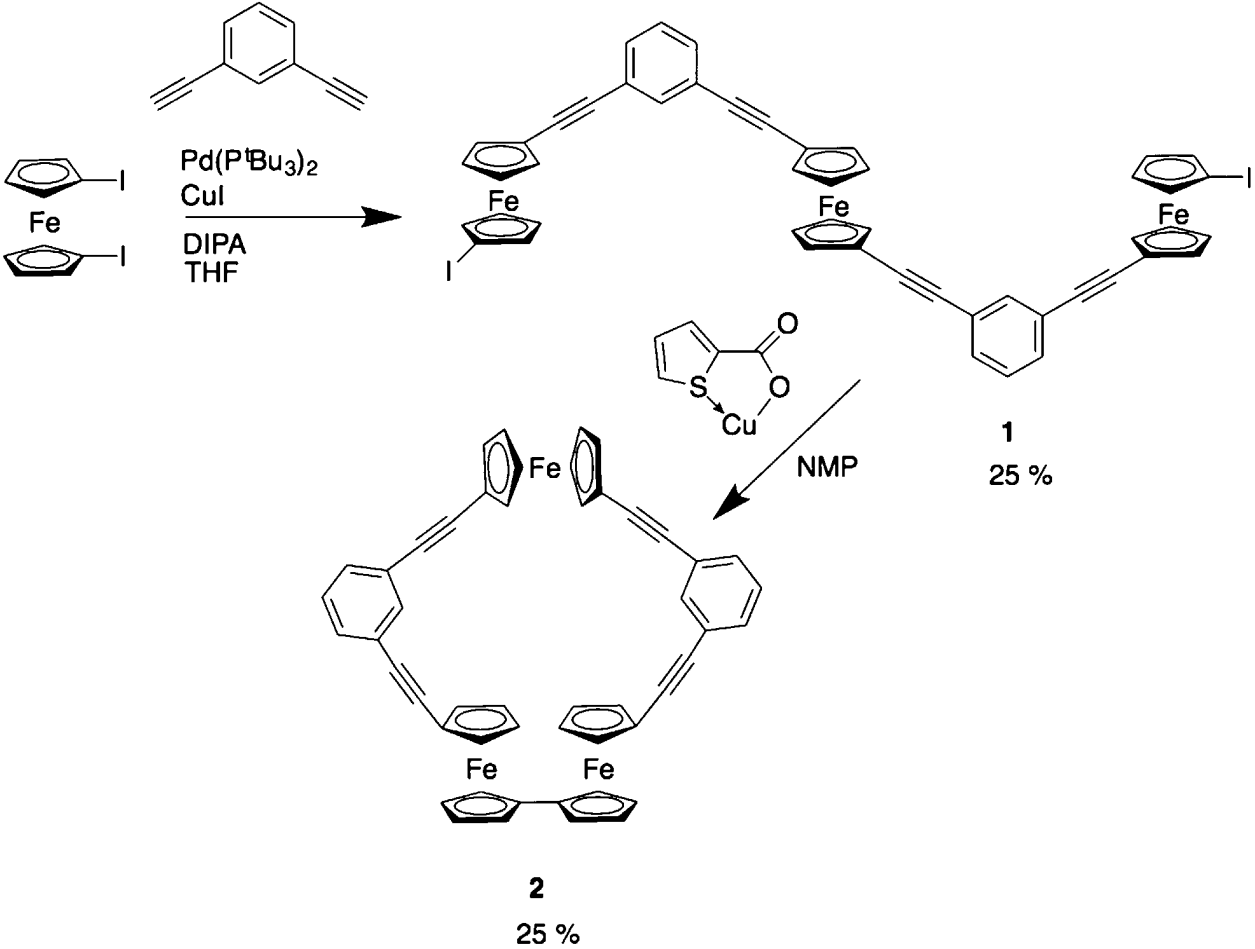
Synthetic route to triferrocene macrocycle **2**.

To form the macrocyclic product **2**, the linear system was cyclized using an Ullmann‐like coupling mediated with copper(I) thiophene‐2‐carboxylate (CuTC). The desired product was achieved by using high‐dilution conditions, a two‐day reaction time, and isolated in a yield of 25 % after chromatographic purification. The product has been identified by NMR, EA, mass spectrometry, and X‐ray crystallography (see the Supporting Information).

Following the success with the synthesis of **2**, a symmetrical molecule with two sets of the biferrocene motif (compound **7**) was proposed. After several attempts at different synthetic routes, the procedure shown in Scheme 2 was established. This step‐wise method allows control in the build‐up of the molecule and in the final cyclisation step. The method uses the same Sonogashira and Ullmann‐like coupling methods as utilized in the synthesis of **2**. Each intermediate and the final product was isolated through either column chromatography or recrystallization and fully characterized (see the Supporting Information) but compounds **5**–**7** were only sparingly soluble.

**Scheme 2:**
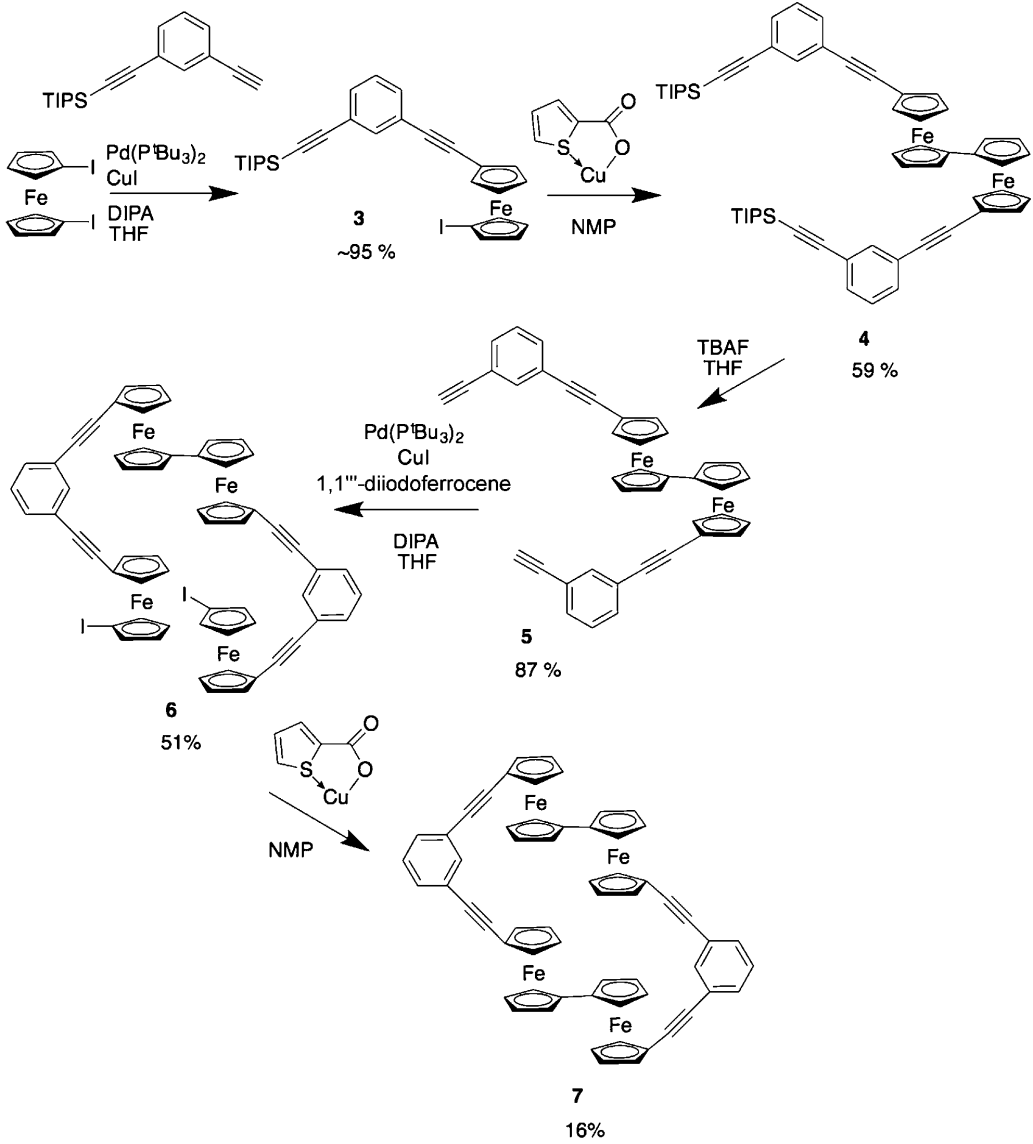
Synthetic route to tetraferrocene macrocycle **7**.

The ^1^H NMR spectra of the cyclic products **2** and **7** (Figure 1) show the protons of the phenyl ring at downfield shifts and the pseudo triplets of the ferrocenes at a higher field. The decrease in quantity of pseudo triplets in the ^1^H NMR spectrum of **7** are due to the increase in symmetry and indicates the equivalency of all the ferrocene units. The ferrocene peaks in **2** can be identified (from left); the first and third are associated with the singular ferrocene while the second, fourth, fifth, and sixth are related to the biferrocene unit. Owing to the symmetry of **7,** all Cp−H peaks are related to the equivalent ferrocenes. This was confirmed by a 2D NMR spectrum. Conventional ^1^H NMR spectra are displayed and annotated in Figure 1.

**Figure 1:**
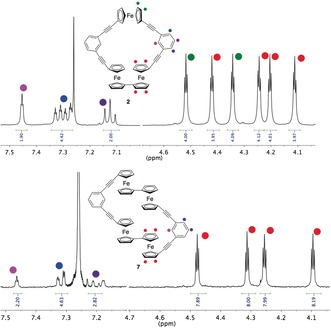
^1^H NMR spectra of **2** and **7** in CHCl_3_ with color indicators to indicate specific proton environments.

The crystal structure of **2** (Figure 2) shows the macrocycle to have adopted an open, slightly folded conformation with a significant cavity (Supporting Information, Figure S9); the angle between the plane consisting of Fe1 and the centroids of the two C_6_H_4_ rings, and that comprising the same two centroids with Fe2 and Fe3 is ca. 19°, though the latter plane is not particularly flat itself being only coplanar to within ca. 0.20 Å. The biferrocene has a twisted *anti* conformation (Fe2⋅⋅⋅Fe3 torsion angle 165.59(2)°, Fe⋅⋅⋅Fe separation 5.0752(6) Å). This linkage also has a noticeable fold deformation, the centroid of one Cp ring lying circa 0.24 Å out of the plane of the other. The Fe1⋅⋅⋅Fe2 and Fe1⋅⋅⋅Fe3 separations across the macrocycle are 10.6097(7) and 12.2662(7) Å respectively.

**Figure 2:**
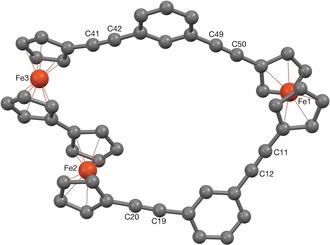
The X‐ray crystal structure of **2**.^39^

Both systems contain multiple redox‐active centers that could communicate through bond or space and may, on oxidation to a mixed valence state, exhibit electronic interactions between the different redox sites that are specific to the cyclic structure. Owing to the low solubility of **7**, electrochemical investigations did not provide conclusive results; for example, at room temperature and in a range of solvents, the voltammograms contained additional features, which are likely related to solubility issues. Running the voltammograms at 60 °C in THF indicated two distinct redox transitions (Supporting Information, Figure S11) but degradation was observed at high potentials. The electrochemical properties were thoroughly examined for **2**, through cyclic voltammetry (CV) and differential pulse voltammetry (DPV) in CH_2_Cl_2_, using 0.1 m [*n*Bu_4_N][PF_6_] as the supporting electrolyte. As shown in Figure 3, there are three separate redox events. Half‐wave potentials (*E*_1/2_) and peak splittings (Δ*E*) for each redox event are shown in Table 1. For an ideal reversible one‐electron transfer process at room temperature and in the limit of linear diffusion, Δ*E*=59 mV and a peak current ratio of the reverse to the forward peak *i*_pc_/*i*_pa_=1 are expected.^29^ Our results show that this is indeed the case, to a good approximation.

**Figure 3:**
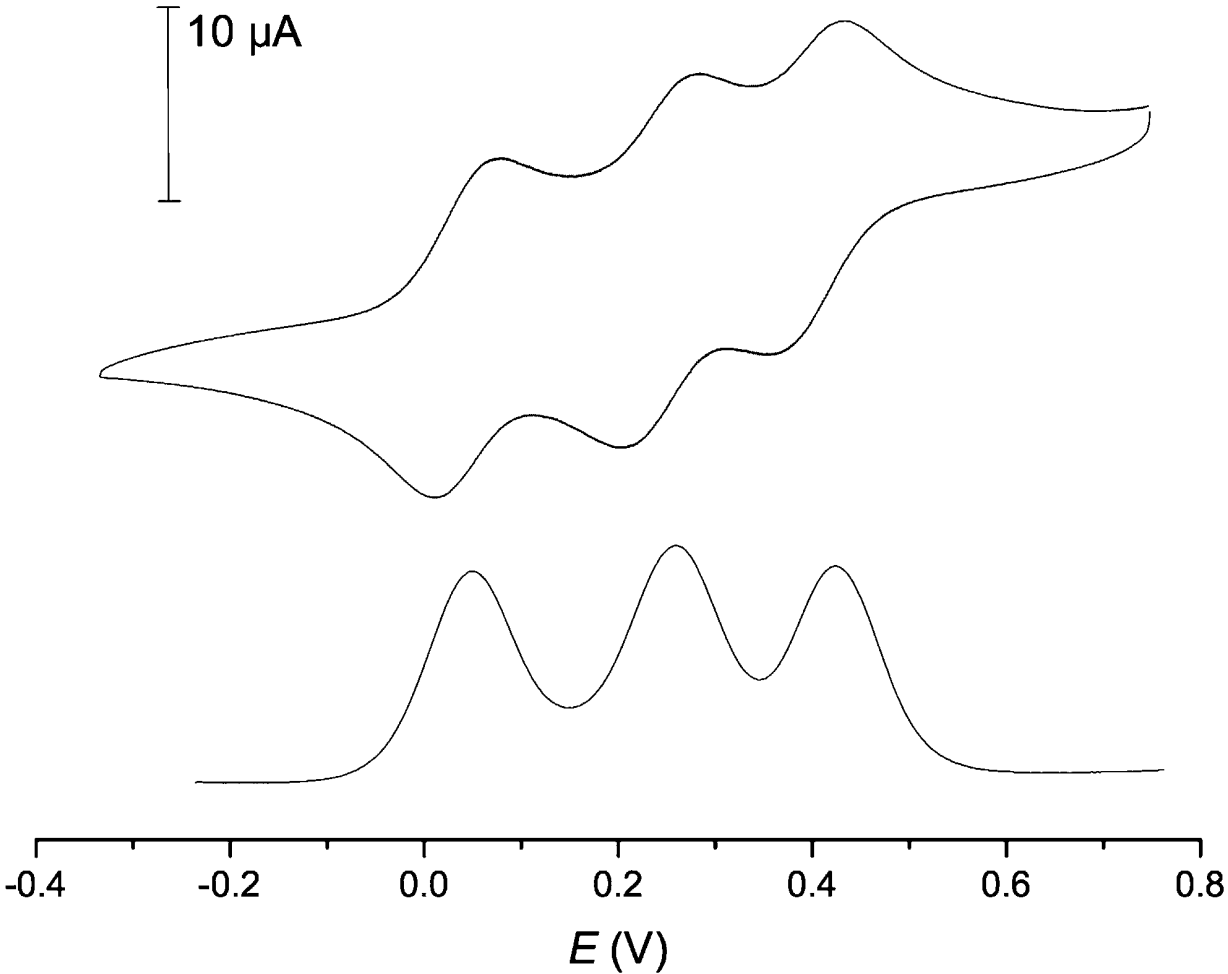
Solution electrochemistry for **2**. Cyclic (top) and differential pulse (bottom) voltammograms recorded in 0.1 m [*n*Bu_4_N][PF_6_]/CH_2_Cl_2_ (*E* vs. [Cp_2_Fe]/[Cp_2_Fe]^+^, corrected for *i*R_s_).

**Table 1:** Electrochemical data for cyclic voltammetry experiments of **2** with 0.1 m [*n*Bu_4_N][PF_6_]/CH_2_Cl_2_.^[a]^

Event	*E*_1/2_	Δ*E*
1st	0.076	0.067
2nd	0.284	0.069
3rd	0.437	0.068

[a] Conditions: scan rate *v*=0.04 V s^−1^; working electrode: glassy carbon; counter and reference electrode: Pt wire; all potentials reported in V relative to an internal [Cp*_2_Fe]/[Cp*_2_Fe]^+^ standard (vs. [Cp_2_Fe]/[Cp_2_Fe]^+^)^30^ and corrected for *i*R_s_.

Owing to the proximity of each of the individual redox events, we found it difficult to reliably define a baseline for some of the redox transitions. However, this is straightforward for the first cathodic (3+/2+ transition) and the first anodic wave (0/1+ transition). The peak current ratio, as defined above, then amounts to 0.97. It was also calculated that the sum of all cathodic peak currents divided by the sum of all anodic peak currents is 1.01 at 0.1 V s^−1^. For both of these peaks, *i*_p_ scales linearly with *v* ^1/2^ (Supporting Information, Figures S14, S15). Combined with the data in Table 1, this suggests that the redox processes involved are indeed reversible and occur in the linear diffusion regime, under the conditions used here (see the Supporting Information for additional electrochemical data, including sweeps at different scan rates; Figures S12 and S13). We did not observe any significant degradation of the redox species, except at the slowest scan rate used (*v*=0.04 V s^−1^) at high potential (above the 2+/3+ transition). Additionally, CVs were run in [*n*Bu_4_N][BAr^F^_4_] solution (BAr^F^_4_=tetrakis[3,5‐bis(trifluoromethyl)phenyl]borate). Owing to the weak ion pairing capabilities of BAr^F^_4_ and increased electrostatic interactions between the redox sites, half‐wave potential splittings were significantly enhanced.^31^, ^32^ This pushes the third oxidation wave close to the anodic stability limit of our electrolyte with concomitant changes in the voltammetric response (Supporting Information, Figure S16).

The close proximity to the first and third redox waves of **2** with those of 1,1′′′′‐bis(ethynylphenyl)biferrocene **8** and of the second wave to that of 1,1′′′‐bis(phenylethynyl)ferrocene **9** (Supporting Information, Figure S17) suggest the corresponding sequence of redox events that is, that the 2nd oxidation of **2** is linked to the singular ferrocene and the first and third correspond to the biferrocene unit.^22^, ^33^ This assignment is also in agreement with the results of our DFT calculations (see below). The cyclic voltammetry data does not give evidence of significant ring or spatial proximity effects owing to the structure of these molecules. Previous reports of oligoferrocenes with similar alkynyl linkers have shown no evidence for electronic communication between individual ferrocene sites through bond, however there has been some evidence of through space electrostatic interaction.^17^, ^25^, ^26^

One indicator of such electronic communication are electronic absorption bands at low energy, typically in the near infrared (NIR), for the mixed‐valence (MV) forms that relate to the transfer of electron density from the reduced to the oxidized ferrocene subunits (intervalence charge‐transfer, IVCT). Such bands were recently observed for the MV states of cyclic sexiferrocene.^2^ With the aim to probe for such IVCT bands, we compared the electronic spectra of **2**, **2^+^**, **2^2+^**, and **2^3+^** in the UV/Vis/NIR range. The mono‐ and di‐cations were generated by chemically oxidizing **2** with one or a slight excess over 2 equiv of acetylferrocenium hexafluorophosphate (*E*_1/2_ =0.27 V), respectively, while **2^3+^** was produced by oxidation of **2** with three equivalents of Ag^+^[SbF_6_]^−^. Their vis/NIR spectra are shown in Figure 4, and relevant data are compiled in Table 2.

**Figure 4:**
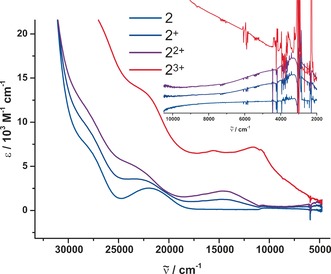
Electronic absorption spectra of **2** in CH_2_Cl_2_ in its various oxidation states.

**Table 2:** UV/Vis/NIR data for **2 n^+^**_._

	UV/Vis/NIR λ [nm] (*ϵ* [L mol^−1^ cm^−1^])	IR/NIR λ [nm] ($\tilde{\nu }$ [cm^−1^]; *ϵ* [L mol^−1^ cm^−1^])
**2**	350 (sh), 455 (2520)	–
**2^+^**	430 (3560), 681 (1250)	2500 (4020; 740), 3176 (3149; 590)
**2^2+^**	430 (4900), 683 (2200)	2459 (4060; 1060), 3175 (3150; 820)
**2^3+^**	440 (13 500), 642 (6800), 860 (7200)	broad, featureless

Stepwise oxidation to **2^+^** and then to **2^2+^** induces a growth of a band at ca. 680 nm (14 700 cm^−1^), which is readily identified as the typical Cp→Fe charge transfer (CT) band of a ferrocenium ion.^34^ Revealingly, the intensity of that band roughly doubles on the second oxidation. The most interesting observation is the appearance of weak, broad absorption features in the NIR. Monitoring the spectra from the IR/NIR side and spectral deconvolution of the NIR region (Table 2; Supporting Information, Figures S18, S19) reveals that the overall NIR absorption is composed of a stronger and broader band at ca. 2500 nm (Δ$\tilde{\nu }$ ${}_{1/2}$ ≈4000 cm^−1^) and a sharper, less intense one at ca. 3175 nm (Δ$\tilde{\nu }$ ${}_{1/2}$ ≈1250 cm^−1^). The observation of that band in **2^+^** and **2^2+^** thus confirms our previous assignment of the first oxidation as based on the biferrocene and that of the second one as involving the remote monoferrocenyl site. More importantly, and despite of the interconnecting π‐conjugated pathway, we have no evidence for an additional IVCT transition between the remote ferrocenyl site and the Fe^III^ of the biferrocenium cation in **2^+^** or the reduced Fe^II^ site of the biferrocenyl and the remote ferrocenium site of **2^2+^**. Tricationic **2^3+^**, however, exhibits a rather intense and broad absoption envelope over the entire Vis/NIR and down to the mid‐IR range (down to 5000 nm, 2000 cm^−1^). The underlying excitations are likely of bridge/Cp‐to‐metal CT origin.

We sought for deeper insight into the electronic structures of **2** and its various oxidized forms by density functional theory (DFT) calculations. Contour plots of the calculated MOs and the respective MO contributions of every subunit as derived from natural bond order (NBO) analysis are compiled in the Supporting Information as Figures S20–S23. Our results indicate that the highest occupied molecular orbital (HOMO) and the HOMO−2 of **2** are largely centered on the biferrocene (bfc) moiety whereas the HOMO−1, LUMO, and LUMO+1 receive major contributions from the 1,3‐diethynylphenylene bridges along with minor contributions from the remote ferrocene moiety Fc3. Our calculations correctly predict that on the first oxidation the electron is removed from one ferrocenyl subunit (biFc1) of the biferrocene. This follows from the β‐LUSO and the calculated spin density map, as graphically shown in the Supporting Information, Figure S24 and listed in Table S1. Further MOs with major contributions of the bfc unit are found as the α‐LUSO and the LUSO+1 and LUSO+2 of the α‐ and β‐manifolds. Again in agreement with our experiment, the second electron is lost from the remote ferrocenyl site Fc3. Quite interestingly, the spin density at the bfc entity of **2^2+^** is computed to be equally distributed over both biFc sites as opposed to localization at only one Fe site (biFc1) in **2^+^**. This is not necessarily at odds with the experimentally observed near invariance of the IR/NIR spectra to the second oxidation. Biferrocenium cations as they are present in **2^+^** and **2^2+^** are generally situated close to or at the borderline between fully delocalized or valence‐trapped mixed valence systems of Class III or II according to the Robin–Day classification.^35^ Influences tipping the scale to either one side or the other such as the solvent of crystallization, the orientation of the counterion or the conformation of the bfc^+^ at the Cp−Cp linkage can be very subtle and do not cause detectable changes of the NIR spectrum.^36^, ^37^, ^38^ Unfortunately, all our attempts to optimize trication **2^3+^** failed to converge.

To conclude, two new multiferrocene macrocycles have been reported, through analogous stepwise Sonogashira and Ullmann‐like coupling methods. The tri‐ferrocene macrocycle **2** exhibits three reversible oxidations related to the bi/ferrocene components of the system. The biferrocene unit is able to electronically share charge and spin densities whereas the separated ferrocene moiety is virtually decoupled from its neighbors. Weak IVCT transitions for a biferrocenium radical cation were observed for **2^+^** and **2^2+^** and confirm the ordering of redox events as proposed from the comparison of the voltammograms of the individual components of macrocyle **2**. The cavity present in the crystal structure of **2** suggests that these molecules could also be accessible for small neutral and charged guests. Our bespoke synthetic methodology allows for further design of small, cyclic or in‐parallel multi‐metallic systems, to also feature surface‐ligating functionalities, and these investigations are currently underway.

## Conflict of interest

The authors declare no conflict of interest.

## Supporting information

As a service to our authors and readers, this journal provides supporting information supplied by the authors. Such materials are peer reviewed and may be re‐organized for online delivery, but are not copy‐edited or typeset. Technical support issues arising from supporting information (other than missing files) should be addressed to the authors.

Supplementary
