# Supplementary material for: Ferrocene‐ and Biferrocene‐Containing Macrocycles towards Single‐Molecule Electronics
Source: Angew Chem Int Ed Engl. 2017 May 12;56(24):6838–42. doi: 10.1002/anie.201702006 (PMC5499722; doi:10.1002/anie.201702006)
Supplement: Supplementary file 1 — Supplementary [file ANIE-56-6838-s001.pdf]

Angewandte Chemie
Eine Zeitschrift der Gesellschaft Deutscher Chemiker

Supporting Information

Ferrocene- and Biferrocene-Containing Macrocycles towards Single-Molecule Electronics

Lucy E. Wilson, Christopher Hassenrück, Rainer F. Winter, Andrew J. P. White, Tim Albrecht, and Nicholas J. Long**

anie_201702006_sm_miscellaneous_information.pdf

Supporting Information

Contents

1: Experimental Data
2: NMR Spectroscopy
3: X-ray Crystallography
4: Electrochemistry
5: Spectroelectrochemistry

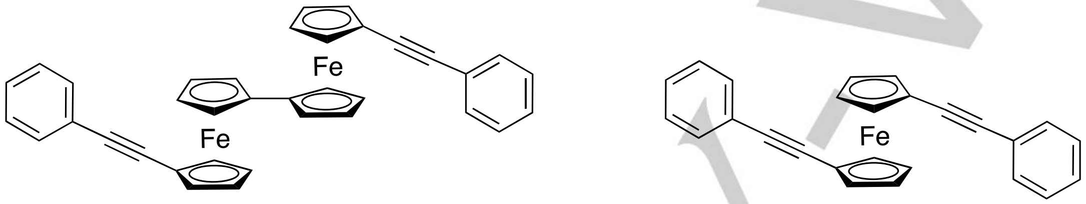

8 **9**

Figure S1: Structures of 1,1'''-bis(phenylethynyl)biferrocene **8** and 1,1'-bis(phenylethynyl)ferrocene **9** discussed within the text.[1,2]

1. Experimental

General: All reactions were performed using standard air sensitive chemistry and Schlenk line techniques under an atmosphere of nitrogen. No special precautions were taken to exclude air during the work-up. Solvents used in reactions were collected from solvent towers sparged with nitrogen and dried with 3 Å molecular sieves, apart from *N*-methyl-2-pyrrolidone (NMP), which was purchased as anhydrous (99.5%) and diisopropylamine (DIPA), which was distilled onto activated 3 Å molecular sieves. 1,1'-Bis(phenylethynyl)ferrocene,[1] 1,1'''-bis(phenylethynyl)biferrocene,[2] 1,1'-diiodoferrocene,[3] copper(I)thiophene-2-carboxylate (CuTC) [4] and 1-(triisopropylsilyl)ethynyl-3-ethynylbenzene[5] were synthesized using literature procedures. All other compounds were purchased from commercial suppliers and used without further purification.
^{1}H and ^{13}C{^{1}H} NMR spectra were recorded on a Bruker Avance 400 or 500 MHz spectrometer and referenced to the residual solvent peaks of $CDCl_3$ at 7.26 and 77.2 ppm respectively or THF-d_8 at 1.72/3.58 ppm and 67.2/25.3 ppm. ^{13}C{^{1}H} spectra were fully assigned where possible using 2D correlation spectroscopy. Coupling constants are measured in Hz. Mass spectrometry analyses were conducted by Lisa Haigh of the Mass Spectrometry Service, Imperial College London. Microanalyses were carried out at the Science Centre, London Metropolitan University, by Stephen Boyer using a Thermo Scientific (Carlo Erba) Flash 2000 Organic Elemental Analyzer, configured for %CHN. Cyclic voltammograms were recorded under an atmosphere of argon in CH_2Cl_2 / 0.1 M [^{n}Bu$_4$][PF_6] or CH_2Cl_2 / 0.2 M [^{n}Bu$_4$][B{$C_6H_3(CF_3)_2$-3,5] on a CHI760C potentiostat (CH Instruments, Austin, Texas) or a Gamry reference 600^{TM} (Gamry Instruments, Warminter, PA, USA) with a glassy carbon disc as working electrode (diameter = 2.5 mm), and Pt-wire as reference and counter electrodes. Analyte solutions were between 0.1-1 mM. Potentials are reported relative to [Cp_2Fe]$^+$/[Cp_2Fe], measured against internal [Cp^*_2Fe]$^+$/[Cp^*_2Fe] references.
NIR/IR experiments were performed on a Bruker Tensor II FT-IR spectrometer and the UV/vis/NIR experiments were recorded using a combined set up of TIDAS MCS UV/NIR and TIDAS PGS NIR spectrometer by j&m Analytik

AG. All oxidized forms of **2** for UV/vis/NIR and NIR/IR spectroscopy were synthesized directly prior to the measurements. A stock solution of **2** in dichloromethane was oxidized with a stock solution of acetylferrocenium hexafluorophosphate in dichloromethane. One or a slight excess to two equivalents of oxidant were used to generate the monocation or dication, respectively. The resulting oxidized species were diluted with dichloromethane to the same concentration. The trication was oxidized using an excess of silver hexafluorophosphate and the resulting solution was filtered by syringe to remove the precipitated metallic silver after adjustment to the same concentration using dichloromethane.
DFT calculations employed the GAUSSIAN 09 program package. Geometry optimizations were performed without any symmetry-constraints. Electronic transitions were calculated by the time-dependent DFT (TD-DFT) method. Within G09 calculations the quasi-relativistic effective core pseudopotentials and the corresponding optimized set of basis functions for Fe were used.[6] Polarized triple ζ basis sets (6-31 G(d), geometry optimization) were employed for structure optimization and Time Dependent-DFT for the molecular orbitals together with the PBE0 functional.[7] The solvent was described by the polarizable continuum calculation model (CPCM) in TD-DFT calculations.[8]

Synthesis of (μ-1,3-Ph)(C≡C-[Fc]-(1,3-Ph)-[Fc]-I)$_2$ (1) : 1,1'-Diiiodoferrocene (7.00 g, 15.91 mmol) in dry DIPA (5 ml) was degassed under N_2 for 10 min. This solution was transferred into a flask containing CuI (48 mg, 0.25 mmol) and 1,3-diethynylbenzene (200 mg, 1.59 mmol) and washed with DIPA (23 ml) and the solution was degassed under N_2 for 10 min. Pd(P^{t}Bu$_3$)$_2$ (20 mg, 0.10 mmol) was added against a flow of N_2 and the reaction was stirred overnight. The solvent was removed and the product was purified by column chromatography (silica, *n*-hexane / DCM [1:0] → [0:1]) to produce (μ-1,3-Ph)(C≡C-[Fc]-(1,3-Ph)-[Fc]-I)$_2$ (**1**) as an orange powder (211 mg, 25%). ^{1}H NMR (400 MHz, $CDCl_3$): δ 7.60 (br s, 2H), 7.42 (dt, $^4J_{H-H}$ = 2.8, $^3J_{H-H}$ = 8.0, 2H), 7.36 (dt, $^4J_{H-H}$ = 1.6, $^3J_{H-H}$ = 8.0, 2H), 7.22 (m, 2H), 4.56 (pseudo-t, $^3J_{\alpha,\beta}$ = 2.0, 4H), 4.48 (pseudo-t, $^3J_{\alpha,\beta}$ = 2.0, 4H), 4.45 (pseudo-t, $^3J_{\alpha,\beta}$ = 2.0, 4H), 4.34 (pseudo-t, $^3J_{\alpha,\beta}$ = 2.0, 4H), 4.27 (pseudo-t, $^3J_{\alpha,\beta}$ = 2.0, 4H), 4.24 (pseudo-t, $^3J_{\alpha,\beta}$ = 2.0, 4H). ^{13}C{^{1}H} NMR (400 MHz, $CDCl_3$): δ 134.3 (2C), 130.8 (2C), 130.7 (2C), 128.5 (2C), 124.2 (2C), 124.1 (2C), 87.9 (2C), 87.8 (2C), 86.4 (2C), 86.2 (2C), 77.4 (2C), 76.5 (4C), 74.2 (4C), 73.2 (4C), 72.3 (4C), 71.2 (4C), 71.1 (4C), 67.5 (2C), 67.0 (2C). MS ES+: m/z 1053.8 ([M+H]$^+$ Calc.: 1053.86) (Found: C, 53.43; H, 2.98. Calc. for $C_{50}H_{32}Fe_3I_2.CH_2Cl_2$ C, 53.71; H, 3.01). υ (cm^{-1}) 2212 (C≡C).

Synthesis of tri-ferrocene macrocycle (2): 1 (158 mg, 0.15 mmol) and NMP (40 ml) were combined under a N_2 environment. CuTC (400 mg, 1.80 mmol) was added against a flow of N_2 and the solution was stirred at RT for 2 days. The solution was filtered through alumina (grade V) with the addition of ethylacetate (50 ml). The solution was washed with brine (5 x 40 ml), dried with $MgSO_4$, filtered and the solvent was removed. The product was purified by column chromatography (silica, n-hexane / DCM [1:0] → [0:1]) to yield the pure product (30 mg, 25%). ^{1}H NMR (400 MHz, $CDCl_3$): δ 7.45 (t, $^4J_{H-H}$ = 1.7, 2H), 7.33 – 7.26 (m, 4H), 7.17-7.11 (m, 2H), 4.52 (pseudo-t, $^3J_{\alpha,\beta}$ = 2.0, 4H), 4.42 (pseudo-t, $^3J_{\alpha,\beta}$ = 2.0, 4H), 4.34 (pseudo-t, $^3J_{\alpha,\beta}$ = 2.0, 4H), 4.24 (pseudo-t, $^3J_{\alpha,\beta}$ = 2.0, 4H), 4.20 (pseudo-t, $^3J_{\alpha,\beta}$ = 2.0, 4H), 4.11 (pseudo-t, $^3J_{\alpha,\beta}$ = 2.0, 4H). ^{13}C{^{1}H} NMR (400 MHz, $CDCl_3$): δ133.8 (2C), 130.5 (4C), 128.2 (2C), 124.3 (2C), 124.1 (2C), 89.0 (2C), 87.3 (2C), 86.7 (2C), 85.8 (2C), 84.8 (2C), 73.9 (4C), 73.3 (4C), 69.8 (4C), 69.7 (4C), 69.6 (4C), 69.0 (4C), 68.2 (2C), 65.9 (2C). MS ES+: m/z 800.0551, ([M]$^+$ Calc.: 800.0552). (Found: C, 74.95; H, 3.94. Calc. for $C_{50}H_{32}Fe_3$ C, 75.04; H, 4.03). IR (ATR): υ (cm^{-1}) 2211 (C≡C).

Synthesis of 1-iodo,1'-(triisopropylsilyl)ethynyl-phenyl-3-ethynylferrrocene (3):
A solution of 1,1'-diiodoferrocene (10.00 g, 22.88 mmol), 1-(triisopropylsilyl)ethynyl-3-ethynylbenzene (1.30 g, 4.60 mmol) and dry THF (30 ml) were degassed with N_2. CuI (33 mg, 0.17 mmol) and DIPA (15 ml) were added to the solution and further degassed. Pd(P^{t}Bu$_3$)$_2$ (70 mg, 0.14 mmol) was added and the solution was stirred overnight, at RT with light removed. The solvent was removed and the product was purified by column chromatography on silica, eluted with *n*-hexane/DCM ([1:0]→[8:2] v/v). The product was collected as an orange oil (1.99 g, 95%).^{1}H NMR (400 MHz, $CDCl_3$): δ 7.63 (s, 1H), 7.47 – 7.41 (m, 2H), 7.29 – 7.25 (m, 1H), 4.48 (pseudo-t, $^3J_{\alpha,\beta}$ = 2.0, 2H), 4.46 (pseudo-t, $^3J_{\alpha,\beta}$ = 2.0, 2H), 4.27 (pseudo-t, $^3J_{\alpha,\beta}$ = 2.0, 2H), 4.24 (pseudo-t, $^3J_{\alpha,\beta}$ = 2.0, 2H), 1.14 (s, 21H). ^{13}C{^{1}H} NMR (400 MHz, $CDCl_3$): δ 134.9 (1C), 131.5 (1C), 131.4 (1C), 128.4 (1C), 124.1 (1C), 124.0 (1C), 106.4 (1C), 91.4 (1C), 87.9 (1C), 86.3 (1C), 76.5 (2C), 74.2 (2C), 72.3 (2C), 71.1 (2C), 67.4 (1C), 41.3 (1C), 18.8 (6C), 11.5 (3C). MS ES+: m/z 592.1, ([M]$^+$ Calc.: 592.07) (Found: C, 58.89; H, 5.44. Calc. for $C_{29}H_{33}FeISi$ C, 58.80; H, 5.61. υ (cm^{-1}) 2213 (C≡C).

Synthesis of 1,1'''-bis(triisopropylsilylethynyl-phenyl-3-ethynyl)biferrocene (4):
3 (1.99 g, 3.35 mmol) and N-methyl-2-pyrrolidone (60 ml) were placed under a N_2 environment and degassed for 10 min. Copper(I) thiophene-2-carboxylate (3.19 g, 16.75 mmol) was added against a flow of N_2 and the solution was stirred for 2 days. The solution was filtered through alumina (grade V) with ethyl acetate, dichloromethane and then hot dichloromethane. Large quantities of solvent were required to dissolve the product. The pure product was obtained as a red solid by recrystallization from DCM / *n*-hexane (913 mg, 59%).^{1}H NMR (400 MHz, $CDCl_3$): δ 7.53

(s, 2H), 7.40 – 7.32 (m, 4H), 7.25 – 7.21 (m, 2H), 4.43 (pseudo-t, $^3J_{\alpha,\beta}$ = 2.0, 4H), 4.27 (pseudo-t, $^3J_{\alpha,\beta}$ = 2.0, 4H), 4.24 (pseudo-t, $^3J_{\alpha,\beta}$ = 2.0, 4H), 4.08 (pseudo-t, $^3J_{\alpha,\beta}$ = 2.0, 4H), 1.15 (s, 42H). ^{13}C{^{1}H} NMR (400 MHz, $CDCl_3$): δ 134.9 (2C), 131.2 (2C), 131.1 (2C), 128.3 (2C), 124.4 (2C), 123.7 (2C), 106.5 (2C), 91.2 (2C), 89.0 (2C), 85.4 (2C), 84.5 (2C), 72.8 (4C), 70.2 (4C), 69.8 (4C), 68.4 (4C), 65.9 (2C), 18.9 (12C), 11.5 (6C). MS ES+: m/z 930.3428, ([M]+ Calc.: 930.34) (Found: C, 74.91; H, 7.07. Calc. for $C_{58}H_{66}Fe_2Si_2$ C, 74.82; H, 7.15). υ (cm^{-1}) 2943 (TIPS), 2865 (TIPS), 2146 (C≡C), 2211 (C≡C).

Synthesis of 1,1'''-bis(ethynyl-phenyl-3-ethynyl)biferrocene (5): 4 (913 mg, 0.98 mmol) was combined with THF under a N_2 environment. Tetra-n-butylammonium fluoride (1.92 ml, 1.03 mmol) was added and the solution was stirred for 2 h with light excluded. The solvent was removed and the product redissolved in THF. The solution was washed with brine, dried with $MgSO_4$, filtered through alumina (grade II) and the solvent was removed (529 mg, 87%). ^{1}H NMR (400 MHz, THF-d_8): δ 7.47 (s, 2H), 7.38 – 7.33 (m, 4H), 7.29 – 7.25 (m, 2H), 4.48 (pseudo-t, $^3J_{\alpha,\beta}$ = 2.0, 4H), 4.24 (pseudo-t, $^3J_{\alpha,\beta}$ = 2.0, 4H), 4.21 (pseudo-t, $^3J_{\alpha,\beta}$ = 2.0, 4H), 4.07 (pseudo-t, $^3J_{\alpha,\beta}$ = 2.0, 4H), 2.45 (s, 2H). ^{13}C {^{1}H} NMR (400 MHz, THF-d_8): δ 135.4 (2C), 132.1 (2C), 131.6 (2C), 129.3 (2C), 125.6 (2C), 123.7 (2C), 91.6 (2C), 90.0 (2C), 85.6 (2C), 85.4 (2C), 83.5 (2C), 79.4 (2C), 73.4 (4C), 70.9 (4C), 70.5 (4C), 69.0 (4C). MS ES+: m/z 618.0731, ([M]+ Calc.: 618.07) (Found: C, 75.08; H, 4.62. Calc. for $C_{40}H_{26}Fe_2.2C_4H_8O$ C, 75.60; H, 5.51). υ (cm^{-1}) 3264 (C≡C-H), 2211 (C≡C).

Synthesis of 1,1'''-bis(1,1'iodoferrocene-ethynyl-phenyl-3-ethynyl)biferrocene (6):
A solution of 1,1'diiodoferrocene (3.70 g, 8.56 mmol), **5** (529 mg, 0.86 mmol) and dry THF (30 ml) were degassed with N_2. CuI (16 mg, 0.09 mmol) and DIPA (20 ml) were added to the solution and further degassed. $Pd(P^tBu_3)_2$ (33 mg, 0.06 mmol) was added and the solution was stirred overnight, at room temperature with light removed. The solvent was removed and the product was purified by repeated column chromatography on a silica column, eluted with *n*-hexane/DCM ([1:0]→[0:1] v/v). The product was collected as a red solid (536 mg, 51%).^{1}H NMR (400 MHz, $CDCl_3$): δ 7.53 (s, 2H), 7.45 – 7.34 (m, 4H), 7.28 – 7.24 (m, 2H), 4.49 (pseudo-t, $^3J_{\alpha,\beta}$ = 2.0, 4H), 4.47 (pseudo-t, $^3J_{\alpha,\beta}$ = 2.0, 4H), 4.44 (pseudo-t, $^3J_{\alpha,\beta}$ = 2.0, 4H), 4.29 – 4.25 (m, 16H), 4.09 (pseudo-t, $^3J_{\alpha,\beta}$ = 2.0, 4H).^{13}C {^{1}H} NMR (400 MHz, $CDCl_3$): δ 134.4 (2C), 130.8 (2C), 130.5 (2C), 128.4 (2C), 124.5 (2C), 124.1 (2C), 89.0 (2C), 87.7 (2C), 86.5 (2C), 85.5 (2C), 84.5 (2C), 76.6 (4C), 74.3 (4C), 72.8 (4C), 72.3 (4C), 71.1 (4C), 70.2 (4C), 69.9 (4C), 68.4 (4C), 67.5 (2C), 65.9 (2C), 41.3 (2C). MS ES+: m/z 1237.8628, ([M]+ Calc.: 1237.89) (Found: C, 58.05; H, 3.17. Calc. for $C_{60}H_{40}Fe_2I_2$ C, 58.20; H, 3.26). υ (cm^{-1}) 2212 (C≡C).

Synthesis of tetra-ferrocene macrocycle (7):
A solution of **6** (290 mg, 0.234 mmol) and NMP (50 ml) was degassed with N_2 for 10 min. CuTC (508 mg, 2.66 mmol) was added against a flow of N_2 to the solution. The reaction was stirred for 2 days, at RT and covered from light. The solution was filtered through alumina (grade V) with THF. The solution was washed with brine, dried with $MgSO_4$ and the solvent removed. The product was gained through multiple recrystallizations in THF/CH_2Cl_2 as a red solid (37 mg, 16 %). ^{1}H NMR (400 MHz, $CDCl_3$): δ 7.46 (s, 2H), 7.33-7.30 (m, 4H), 7.22-7.17 (m, 2H) 4.48 (pseudo-t, $^3J_{\alpha,\beta}$ = 2.0, 4H), 4.32 (pseudo-t, $^3J_{\alpha,\beta}$ = 2.0, 4H), 4.26 (pseudo-t, $^3J_{\alpha,\beta}$ = 2.0, 4H), 4.10 (pseudo-t, $^3J_{\alpha,\beta}$ = 2.0, 4H). ^{13}C {^{1}H} NMR (500 MHz, $CDCl_3$): 130.3 (2C), 128.3 (4C), 124.3 (2C), 72.8 (8C), 70.2 (8C), 69.8 (8C), 68.4 (8C). MS ES+: m/z 984.0561, ([M]+ Calc.: 984.05) (Found: C, 60.78; H, 3.74. Calc. for $C_{60}H_{40}Fe_4.3CH_2Cl_2$, 61.07; H, 3.74). υ (cm^{-1}) 2213 (C≡C).

WILEY-VCH

2. NMR Spectroscopy

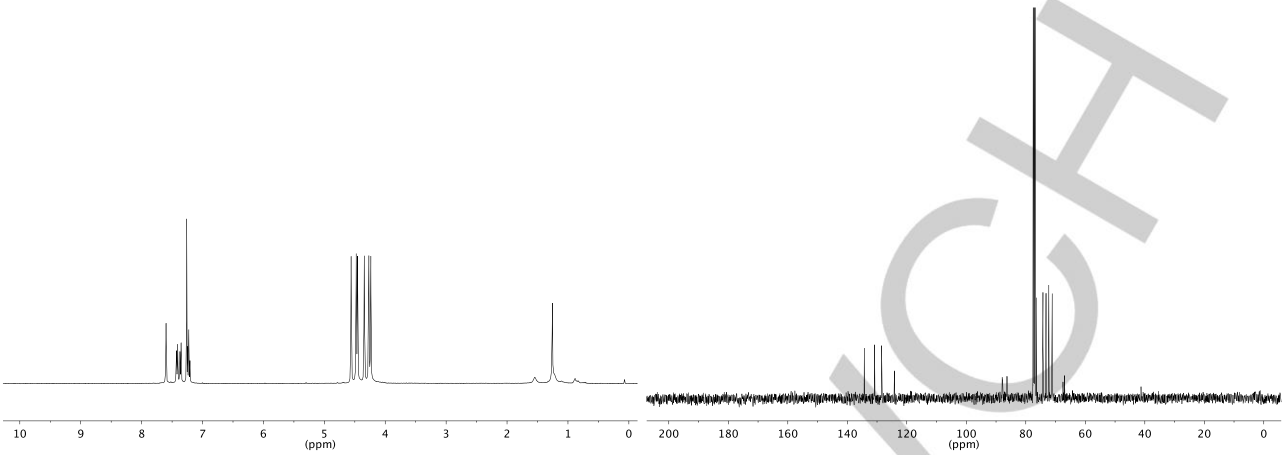

Figure S2: (μ-1,3-Ph)(C≡C-[Fc]-(1,3-Ph)-[Fc]-I)$_2$ **1:** ^{1}H (400MHz, $CDCl_3$) and ^{13}C{^{1}H} NMR (400MHz, $CDCl_3$)

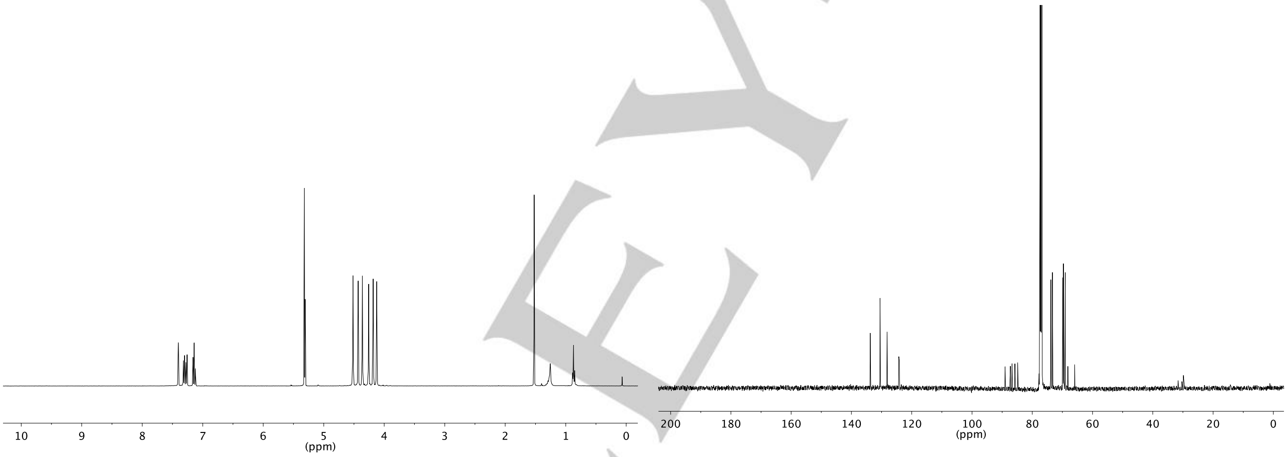

Figure S3: Compound **2:** ^{1}H (400MHz, CD_2Cl_2) and ^{13}C{^{1}H} NMR (400MHz, $CDCl_3$)

WILEY-VCH

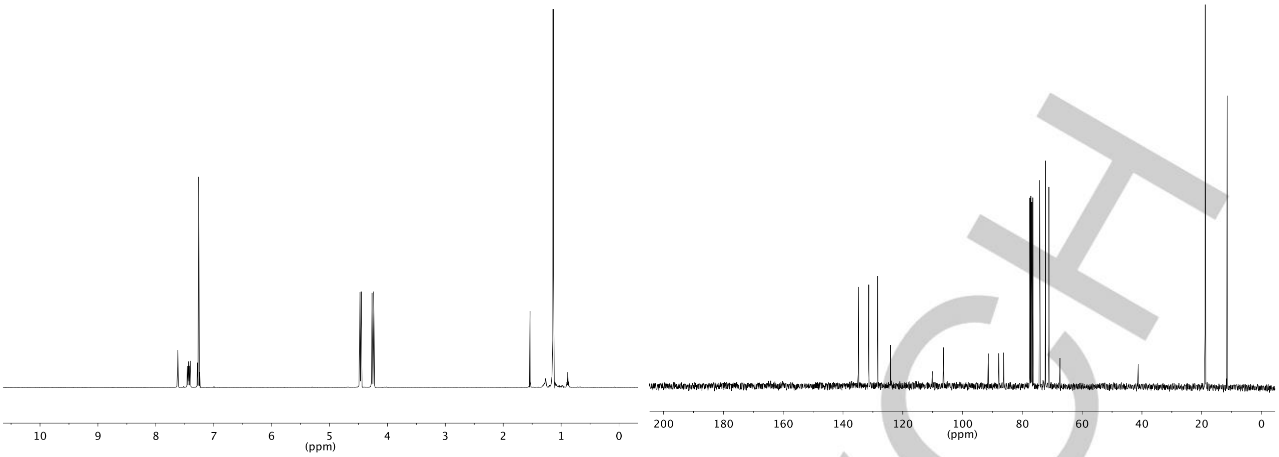

Figure S4: 1-Iodo,1'-(triisopropylsilyl)ethynyl-phenyl-3-ethynylferrrocene **3:** ^{1}H (400MHz, $CDCl_3$) and $^{13}C\{^{1}H\}$ NMR (400MHz, $CDCl_3$)

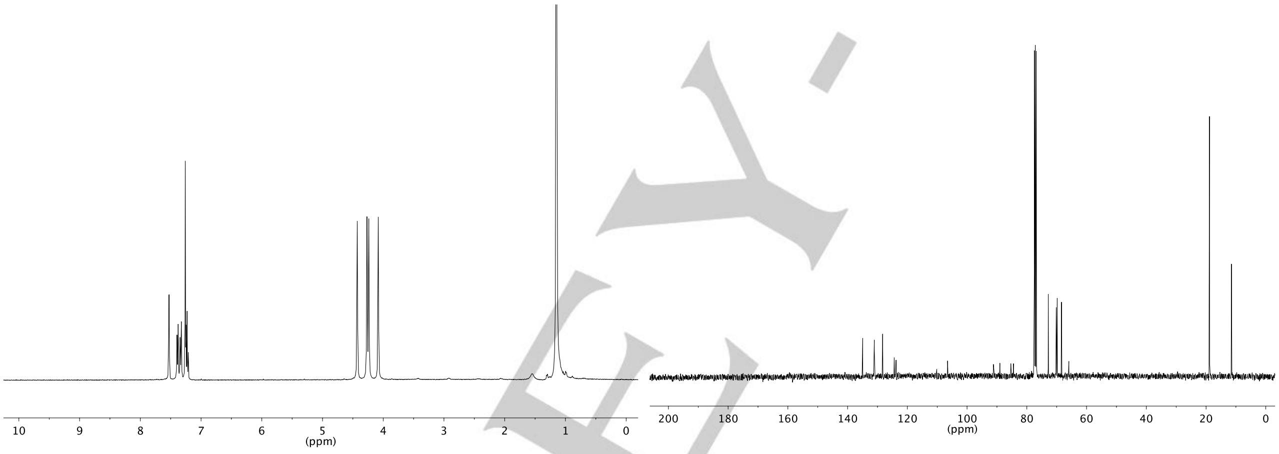

Figure S5: 1,1'''-Bis(triisopropylsilylethynyl-phenyl-3-ethynyl)biferrocene **4:** ^{1}H (400MHz, $CDCl_3$) and $^{13}C\{^{1}H\}$ NMR (400MHz, $CDCl_3$)

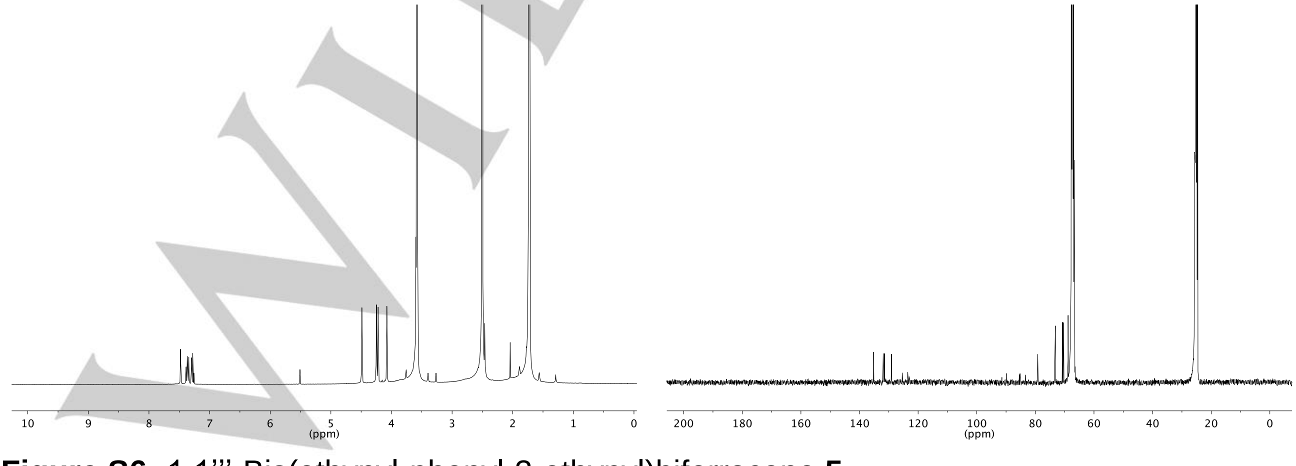

Figure S6: 1,1'''-Bis(ethynyl-phenyl-3-ethynyl)biferrocene **5:**
^{1}H (400MHz, THF-d_8) and $^{13}C\{^{1}H\}$ NMR (400MHz, THF-d_8)

WILEY-VCH

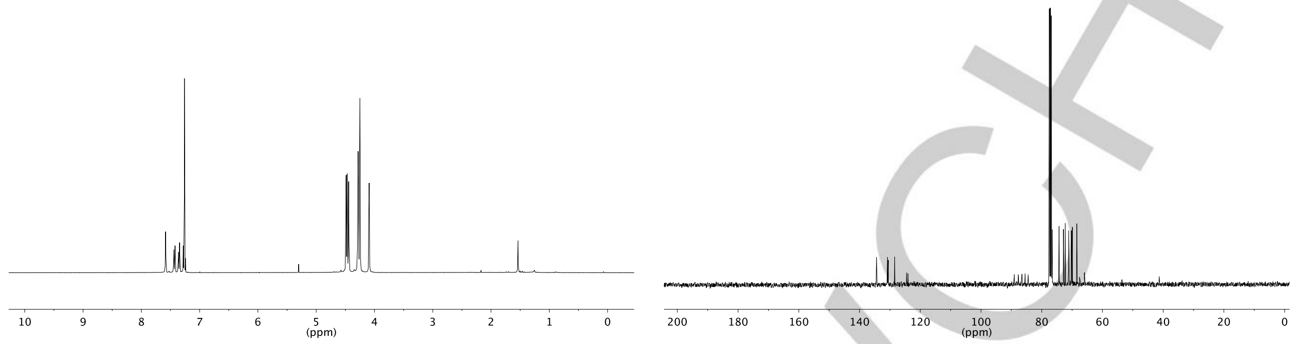

Figure S7: 1,1’’’-Bis(1,1’iodoferrocene-ethynyl-phenyl-3-ethynyl)biferrocene **6:** ^{1}H (400MHz, $CDCl_3$) and ^{13}C{^{1}H} NMR (400MHz, $CDCl_3$)

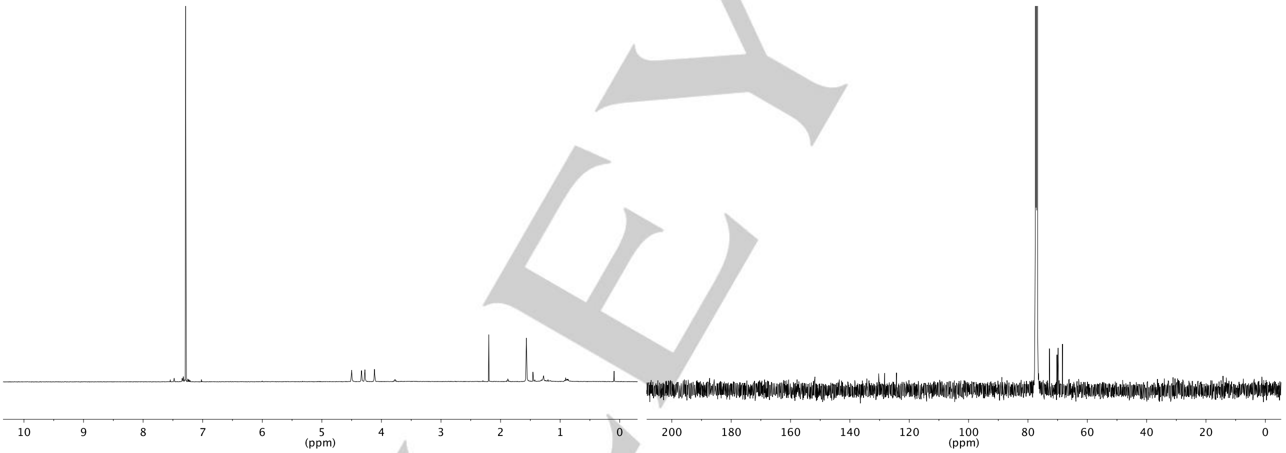

Figure S8: Compound **7:** ^{1}H (400MHz, $CDCl_3$) and ^{13}C{^{1}H} NMR (500MHz, $CDCl_3$)

3. X-ray crystallography

Crystal data for **2**: $C_{50}H_{32}Fe_3 \cdot 1.5(C_6H_{12})$, M = 926.54, monoclinic, $C2/c$ (no. 15), a = 32.7490(7), b = 12.75553(17), c = 21.6508(4) Å, β = 99.3055(19)°, V = 8925.2(3) Å^3, Z = 8, D_c = 1.379 g cm^{-3}, μ(Cu-Kα) = 8.008 mm^{-1}, T = 173 K, orange needles, Agilent Xcalibur PX Ultra A diffractometer; 8811 independent measured reflections (R_{int} = 0.0460), F^2 refinement,[9,10] R_1(obs) = 0.0471, wR_2(all) = 0.1283, 6611 independent observed absorption-corrected reflections [$|F_o| > 4\sigma(|F_o|)$, $2\theta_{full}$ = 135°], 581 parameters. CCDC 1520192.
The C61-based included a cyclohexane solvent molecule in the structure of **2** which was found to be disordered across a centre of symmetry, and two unique orientations of *ca*. 28 and 22% occupancy were identified (with the action of the inversion centre generating two further orientations of the same occupancies). The geometries of both orientations were optimized, the thermal parameters of adjacent atoms were restrained to be similar, and all of the atoms were refined isotropically.

WILEY-VCH

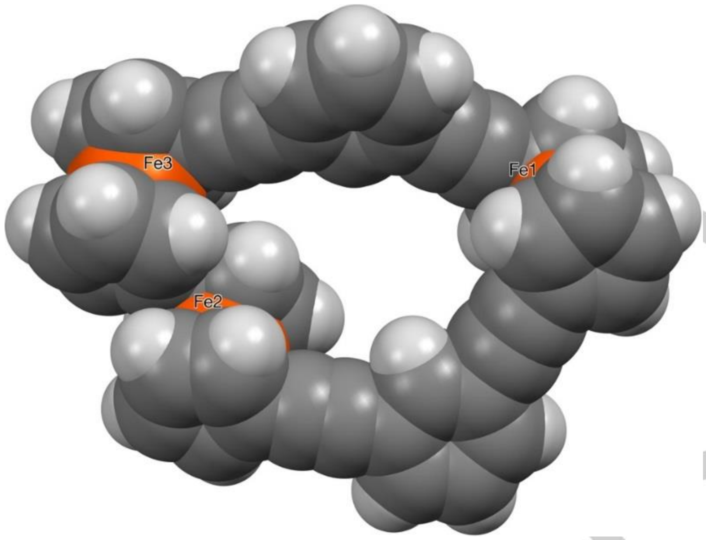

Figure S9: Space filling representation of the X-ray crystal structure of **2**.

WILEY-VCH

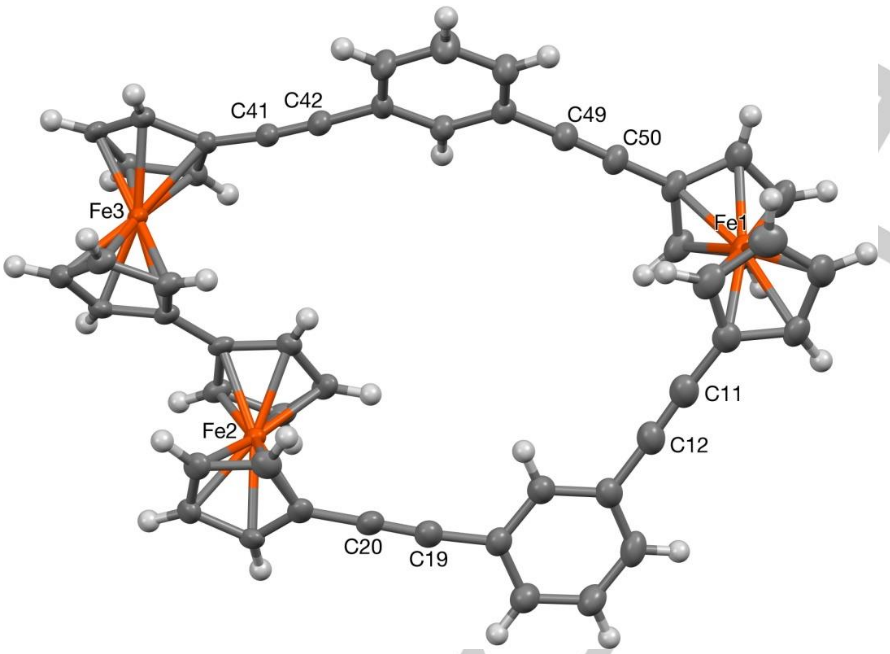

Figure S10: The X-ray crystal structure of **2** (50% probability ellipsoids).

3. Electrochemistry

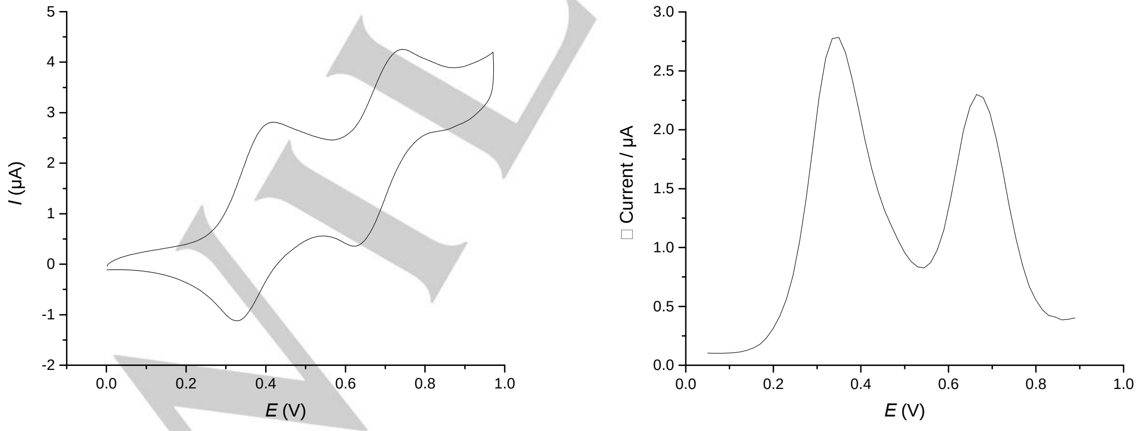

Figure S11. Solution electrochemistry for **7** at scan rate 0.02 V/s in 0.1 M [$^{n}Bu_4N$][BAr^{F}_4]/THF at 60°C (Corrected for iR_s)

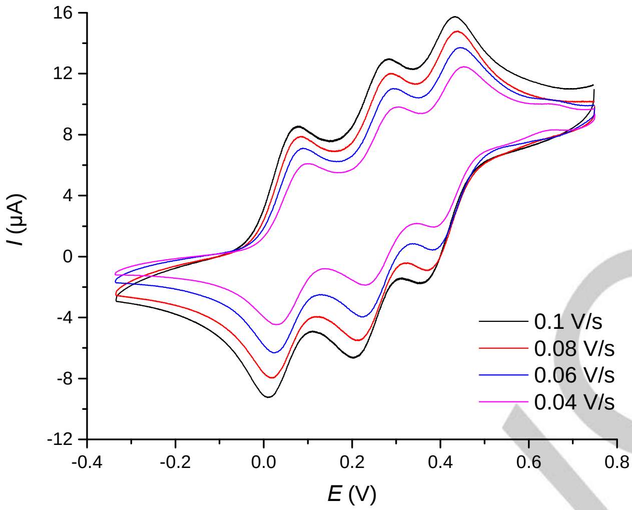

Figure S12. Solution electrochemistry for **2** at scan rates 0.04 V/s – 0.1 V/s in 0.1 M [^{n}Bu$_4$N][PF$_6$]/CH$_2$Cl$_2$ (*E* vs. [Cp$_2$Fe]/[Cp$_2$Fe]$^+$, corrected for *i*R$_s$)

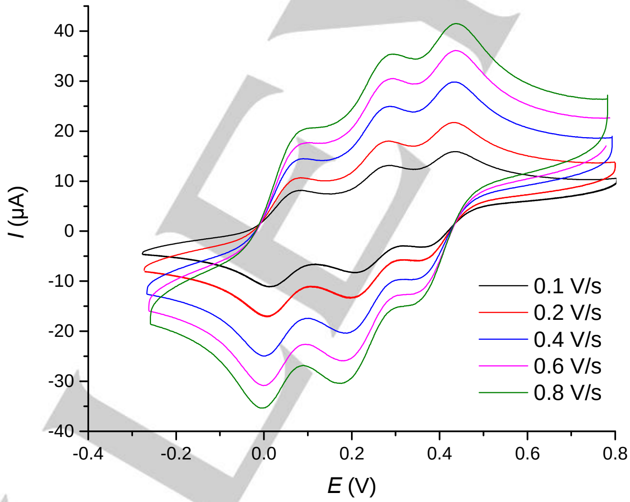

Figure S13. Solution electrochemistry for **2** at scan rates 0.1 V/s – 0.8 V/s in 0.1 M [^{n}Bu$_4$N][PF$_6$]/CH$_2$Cl$_2$ (*E* vs. [Cp$_2$Fe]/[Cp$_2$Fe]$^+$, corrected for *i*R$_s$)

WILEY-VCH

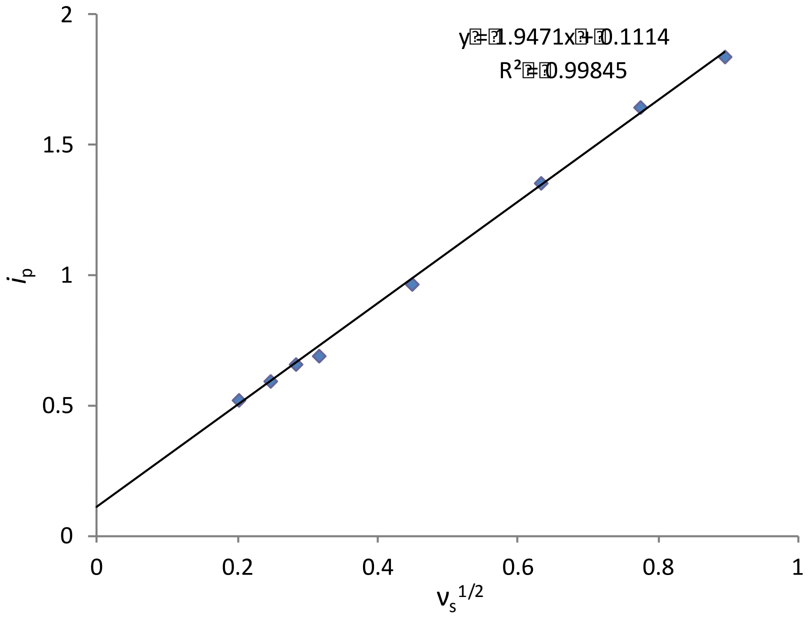

Figure S14: Plot of $i_{pa} \propto v_s^{1/2}$ for 1st peak of **2**

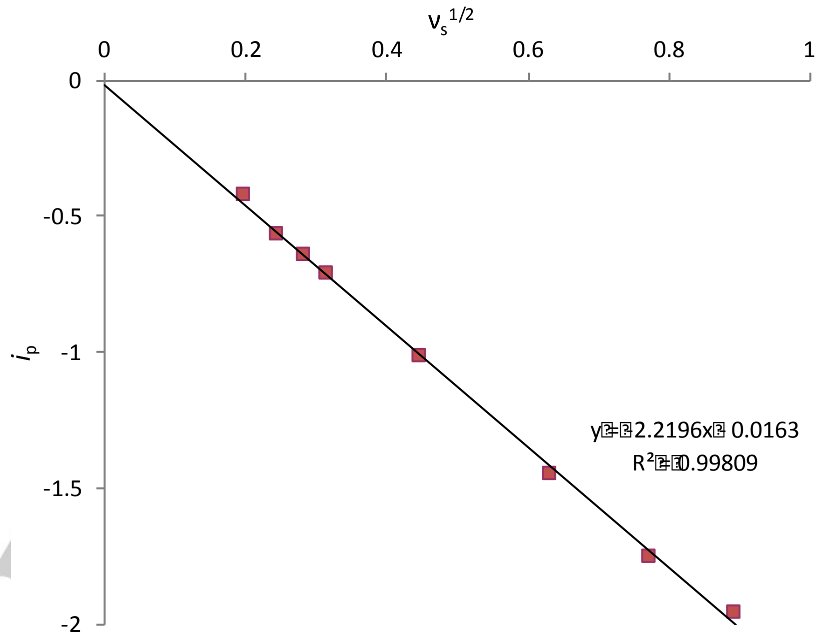

Figure S15: Plot of $i_{pc} \propto v_s^{1/2}$ for 3rd peak of **2**

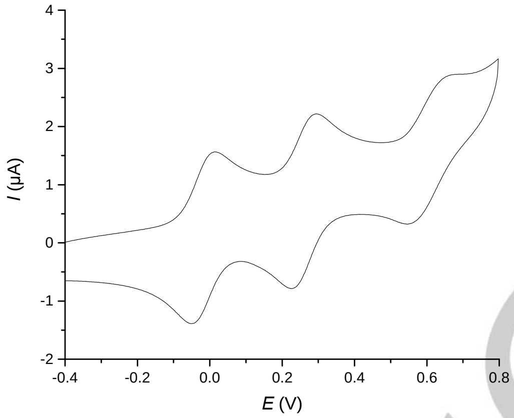

Figure S16: Solution electrochemistry of **2** in 0.2 M [^{n}Bu$_4$N][BAr$^F_4$]/CH_2Cl_2 (*E* vs. [Cp_2Fe]/[Cp_2Fe]$^+$)

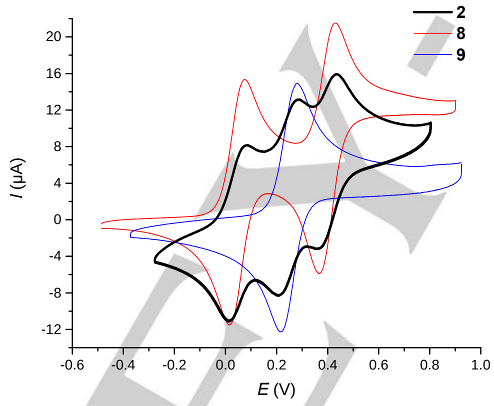

Figure S17: Solution electrochemistry for **2, 8** and **9** in 0.1M [^{n}Bu$_4$N]PF_6/CH_2Cl_2 (*E* vs. [Cp_2Fe]/[Cp_2Fe]$^+$, corrected for *i*R_S). **8** and **9** are portrayed in Figure S1 of the SI.

5. Spectroelectrochemistry

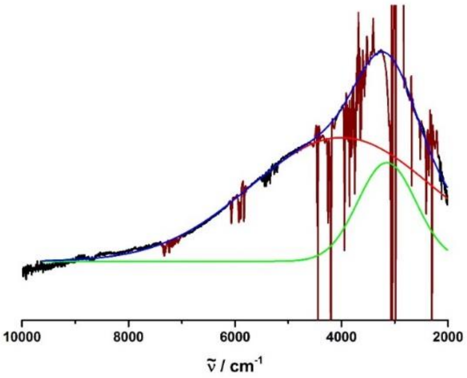

Figure S18: Deconvolution of the electronic NIR transitions in **2$^+$**

WILEY-VCH

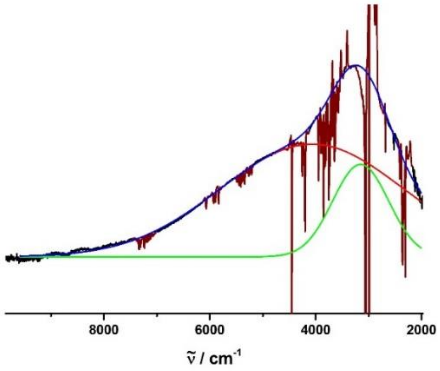

Figure S19: Deconvolution of the electronic NIR transitions in **2^{2+}**

6. DFT data

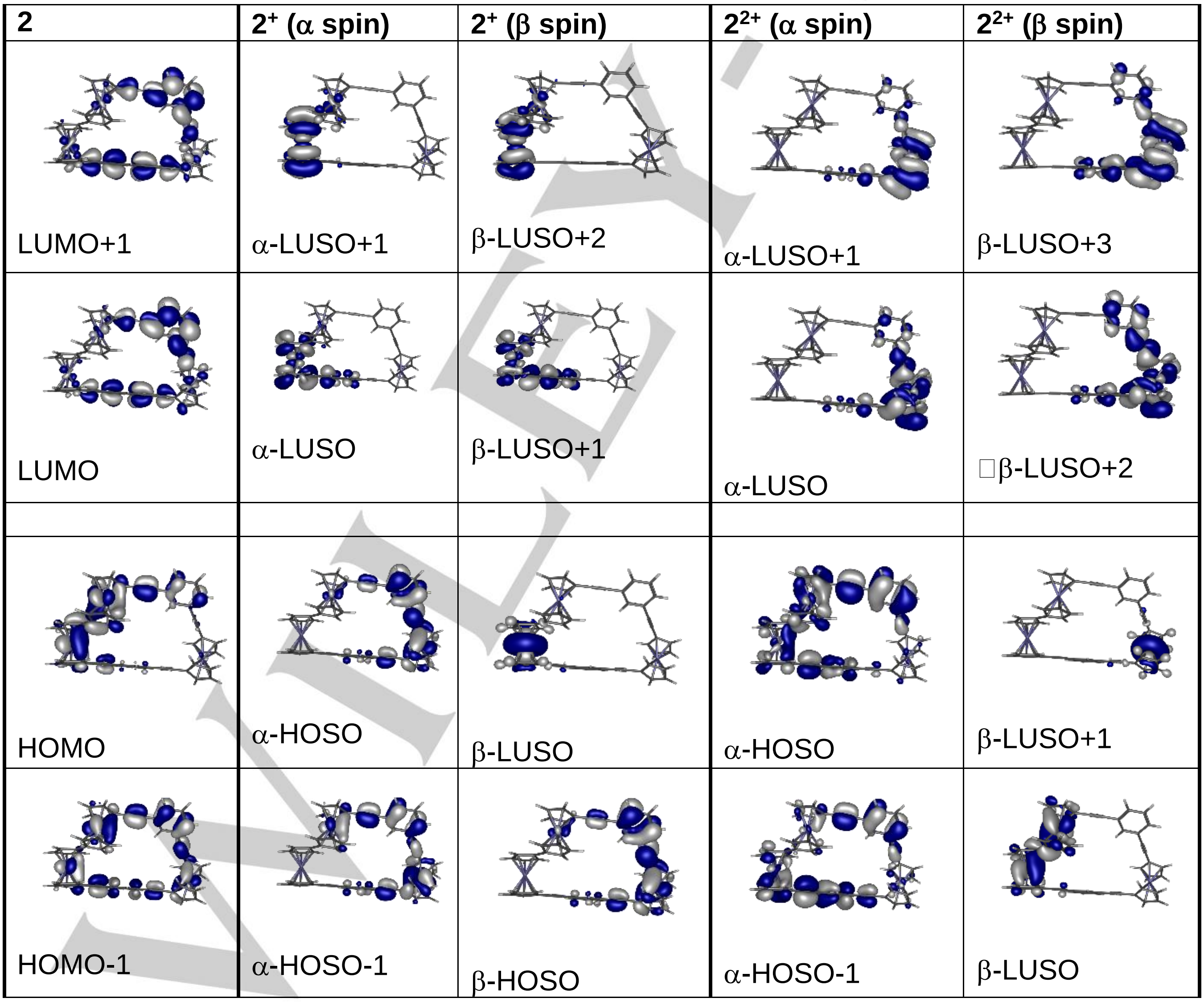

Figure S20: Selected MOs in the frontier MO region of **2**, **2^+**, and **2^{2+}**.

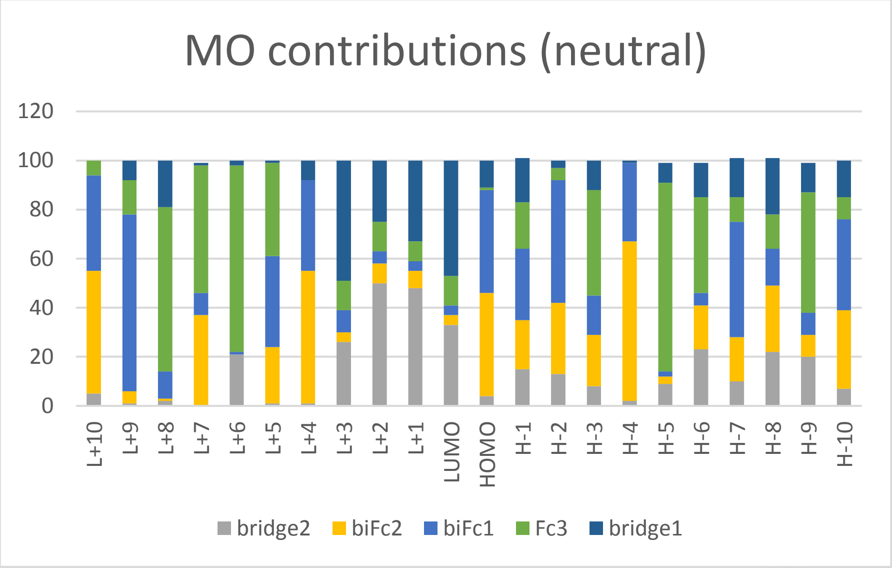

Figure S21: Calculated compositions of selected MOs of **2**

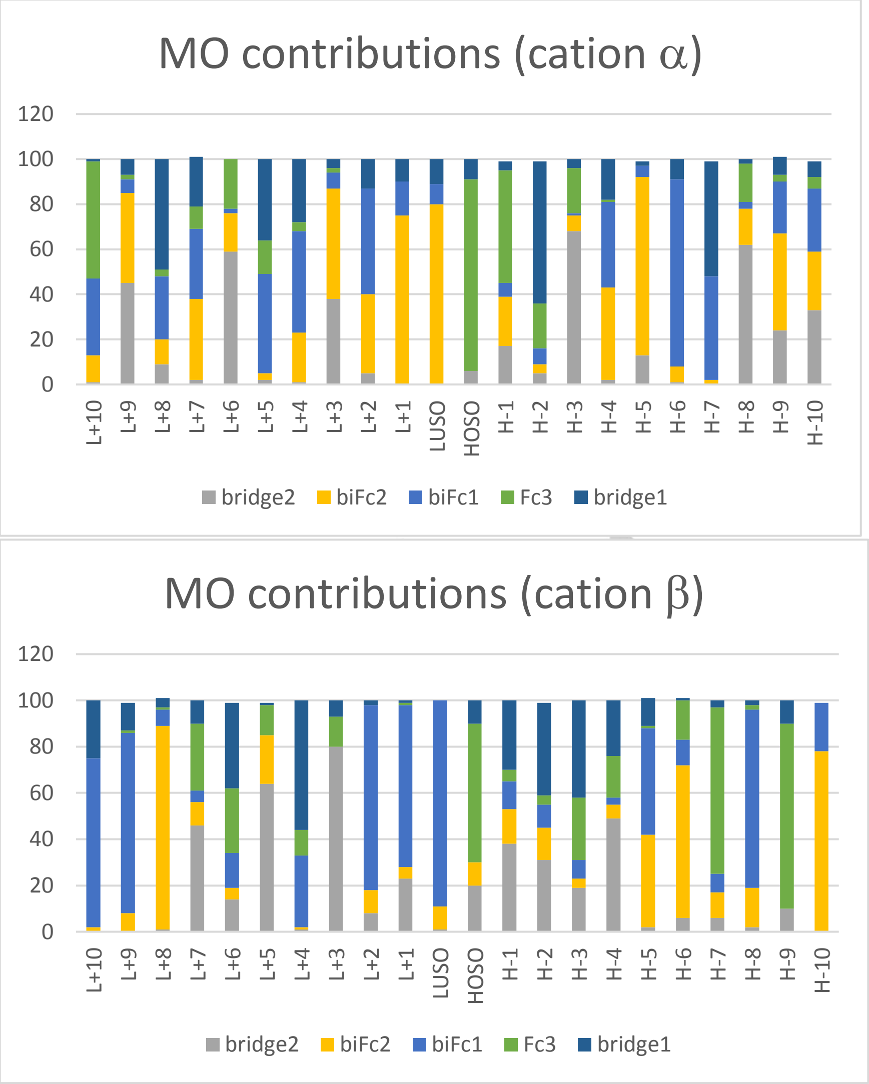

Figure S22: Calculated compositions of selected MOs of $\mathbf{2}^{+}$

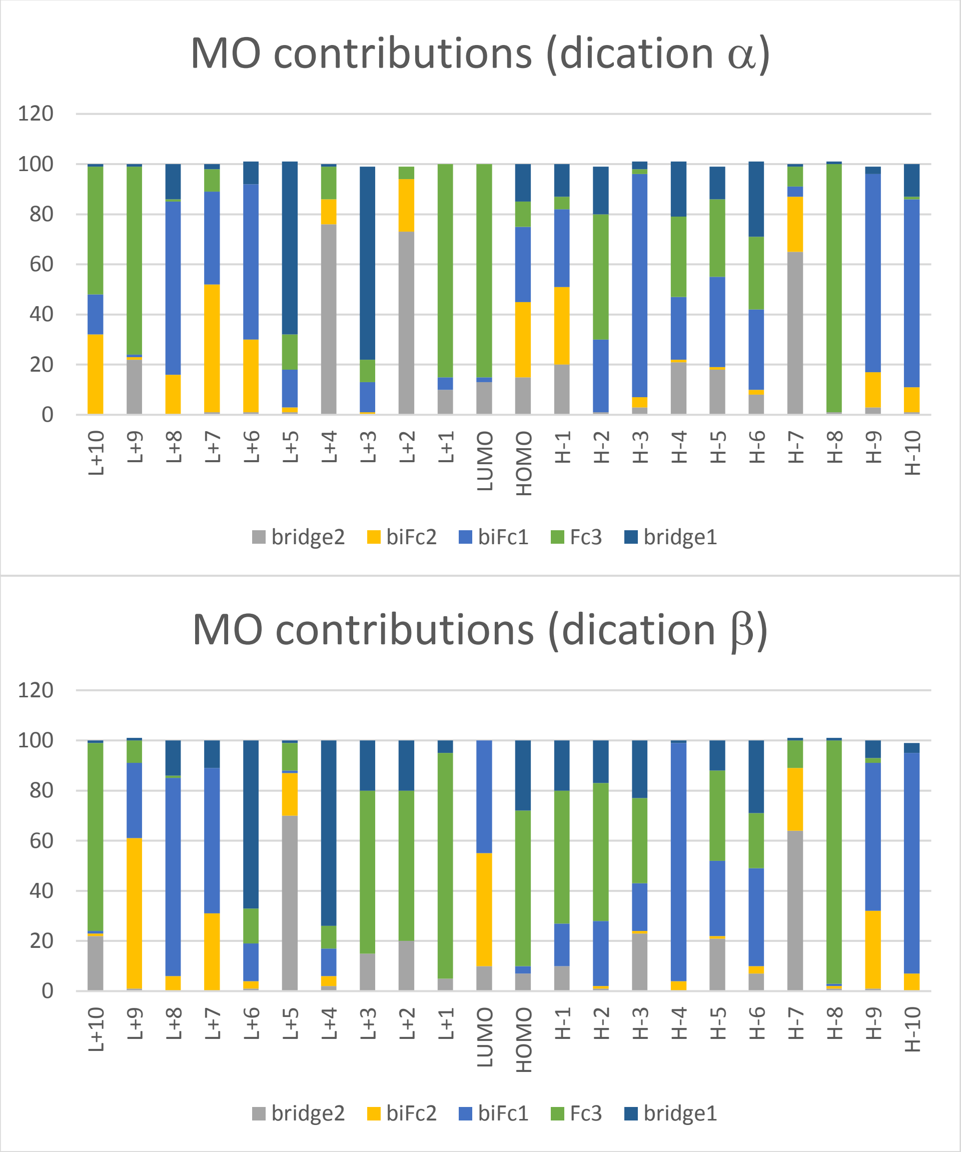

Figure S23: Calculated compositions of selected MOs of **2**$^{2+}$ in the *S* = 1 state

Table S1. Charges and spin densities of individual components of **2**$^{n+}$ as derived from NBO analysis.

Natural Bond Analysis with charge and spin densities

	neutral	cation		dication		0 to 1+	1+ to 2+	1+ to 2+
	charge	charge	spin density	charge	spin density	difference1	difference2	Spin density difference
**biFc1**	0.008	0.032	-0.013	0.492	0.509	0.024	0.459	0.522
**biFc2**	0.008	0.930	1.022	0.487	0.504	0.922	-0.443	-0.517
**Fc3**	0.002	0.006	0.000	0.932	1.019	0.003	0.926	1.019
**bridge1**	-0.010	0.002	-0.001	0.046	-0.015	0.012	0.044	-0.014
**bridge2**	-0.008	0.030	-0.009	0.044	-0.017	0.038	0.014	-0.009
**Sum**	0.000	1.000	1.000	2.000	2.000	1.000	1.000	1.000

WILEY-VCH

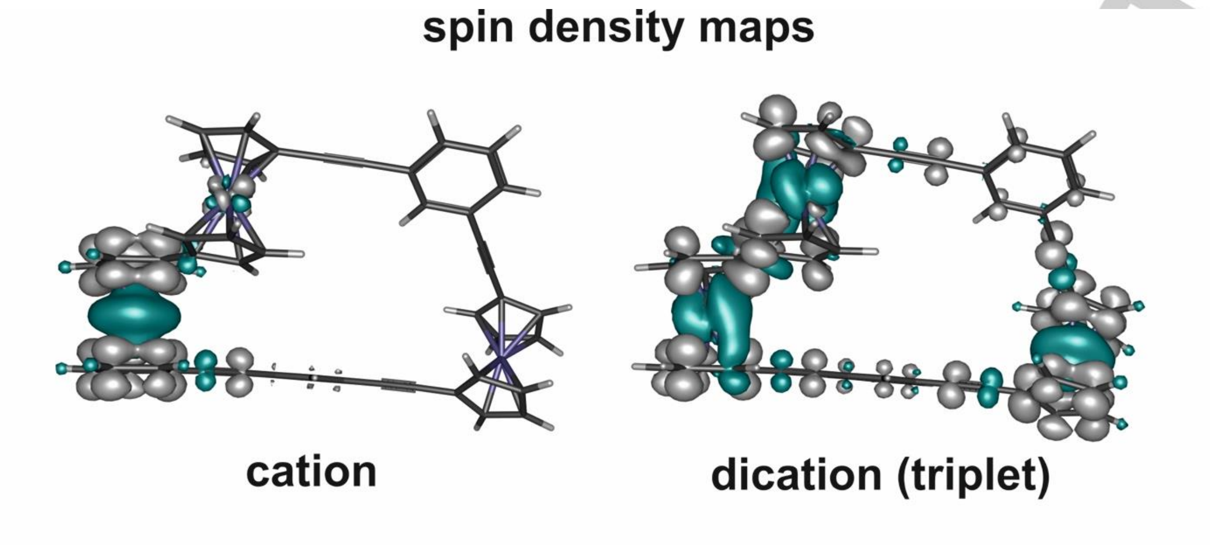

Figure S24: Spin density maps for **2**$^{+}$ in the S = ½ and **2**$^{2+}$ in the S = 1 state

References:

[1] S. L. Ingham, M. S. Khan, J. Lewis, N. J. Long, P. R. Raithby, *J. Organomet. Chem.* **1994**, *470*, 153.
[2] L. E. Wilson, C. Hassenrück, R. F. Winter, A. J. P. White, T. Albrecht, N. J. Long, *Eur. J. Inorg. Chem.* **2017**, *2017*, 496.
[3] M. S. Inkpen, S. Scheerer, M. Linseis, A. J. P. White, R. F. Winter, T. Albrecht, N. J. Long, *Nat. Chem*. **2016**, *8*, 825.
[4] S. Zhang, D. Zhang, L. S. Liebeskind, *J. Org. Chem.* **1997**, *62*, 2312.
[5] S. Y.-L. Leung, A. Y.-Y. Tam, C.-H. Tao, H. S. Chow, V. W.-W. Yam, *J. Am. Chem. Soc.* **2012**, *134*, 1047.
[6] D. Andrae, U. Häußermann, M. Dolg, H. Stoll, H. Preuß, *Theor. Chim. Acta* **1990**, *77*, 123.
[7] J. P. Perdew, K. Burke, M. Ernzerhof, *Phys. Rev. Lett.* **1996**, *77*, 3865.
[8] M. Cossi, N. Rega, G. Scalmani, V. Barone, *J. Comput. Chem.* **2003**, *24*, 669.
[9] SHELXTL, Bruker AXS, Madison, WI, **n.d.**
[10] G. M. Sheldrick, *Acta Crystallogr. Sect. C Struct. Chem.* **2015**, *71*, 3.

Author Contributions

N.J.L, T.A. and L.E.W. conceived the work and designed the experiments. L.E.W. synthesised the materials and performed the solution electrochemical measurements. A.J.P.W. performed the X-ray crystallographic experiments. C.H. and R.F.W. performed the spectroelectrochemical experiments and the DFT calculations. All authors contributed to writing the paper.
